# Mechanochemical Effects on the Synthesis of Copper Orthophosphate and *cyclo-*Tetraphosphate Bulks by the Hydrothermal Hot Pressing Method 

**DOI:** 10.3390/ma2010001

**Published:** 2009-01-09

**Authors:** Hiroaki Onoda, Ken-ichi Okumoto, Atsushi Nakahira, Isao Tanaka

**Affiliations:** 1 Department of Informatics and Environmental Sciences, Faculty of Life and Environmental Sciences, Kyoto Prefectural University, 1-5, Shimogamo Nakaragi-cyo, Sakyo-ku, Kyoto 606-8522, Japan; 2 Taihei Chemical Industrial Co., Ltd., 1-1 Takayasu, Ikaruga-cyo, Nara 636-0104, Japan; 3 Department of Materials Science, Biomaterial Group, Faculty of Engineering, Osaka Prefecture University, Gakuencyo 1-1, Sakai 599-8531, Japan; 4 Department of Materials Science and Engineering, Faculty of Engineering, Kyoto University, Yoshida-Honmachi, Sakyo-ku, Kyoto 606-8501, Japan

**Keywords:** Mechanochemical effects, Copper phosphates, Hydrothermal hot pressing

## Abstract

Copper orthophosphate, Cu_3_(PO_4_)_2_, and *cyclo*-tetraphosphates, Cu_2_P_4_O_12_, were synthesized using phosphoric acid and basic copper carbonate, and then treated with a planetary mill for up to 360 minutes. The un-milled and milled samples were characterized by X-ray diffraction (XRD) and Fourier transform infrared (FT-IR) spectroscopy. SEM images, particle size distribution, specific surface area, UV-Vis reflectance spectra were also used to evaluate the materials. The un-milled and milled materials were used to fabricate copper phosphate bulks by a hydrothermal hot pressing method. The influence of powder condition on the sintering behavior of the copper phosphates was studied.

## 1. Introduction

Phosphates have been widely used as ceramic materials, catalysts, adsorbent, fluorescent materials, dielectric substances, metal surface treatment, fertilizer, detergent, food additives, fuel cells, pigments, etc. Phase transformation of phosphates is likely to occur in hydrolysis and dehydration reactions at elevated temperatures [1,2,3,4]. Typical phosphates include polyphosphate, *cyclo*-phosphate, and ultraphosphate. Polyphosphate has a chain structure in which the PO_4_ unit shares two oxygen atoms; while *cyclo*-phosphate has a cyclic structure; and ultraphosphate has a network structure. 

For different applications, phosphates can be in forms of powders, bulks, or thin layers. Because phosphates decompose to oxide at high temperatures via loss of P_2_O_5_, it is difficult to obtain phosphate bulks by standard sintering techniques. As a novel synthetic process, the hydrothermal hot pressing method has been studied [5,6,7,8,9,10,11]. In this method, mixtures of starting powder and a small amount of water are sintered at relatively low temperatures, which usually leads to porous phosphate materials. The merit of this method is the lower sintering temperature and porous bulk. Porous materials can therefore used as a catalyst, an adsorbent, and so on [12,13]. However, porous phosphate bulks formed in hydrothermal hot pressing are often physically too weak. It is therefore necessary to increase the filling factor, the ratio of real and theoretical density, when using hydrothermal hot pressing process. 

The reforming of solid surface of powder materials gives rise to higher functional properties. Various methods can be used for the purpose of reforming, coating, topochemical method, mechanical treatment, ultraviolet irradiation, etc. The physical and chemical properties of solid materials can be changed by crushing, pressing, milling, and other mechanical treatments. These mechanochemical effects are known to bring about an increase of specific surface area, defects and strain, and the cleavage of chemical bonds, and so on. For these effects, mechanically treated materials are regarded as being in an active state [14,15]. Because of their small particle size, milled powders should be easily sintered at lower temperatures. It is difficult to sinter phosphate materials due to the volatilization of P_2_O_5_. Similarly, phosphate bulks have also been obtained by using the hydrothermal hot pressing process, however they have poor mechanical strength [16]. In this work, we attempt to use mechanochemical treatment to improve the sintering properties of phosphate materials.

Copper orthophosphate and *cyclo*-tetraphosphate were synthesized using phosphoric acid and basic copper carbonate. The obtained phosphates were milled with a planetary-mill. The milled and un-milled materials were examined by X-ray diffraction (XRD) and Fourier transform infrared (FT-IR) spectroscopy. Furthermore, the samples were also characterized using specific surface area analysis, scanning electron microscope (SEM), and particle size distribution, UV-Vis reflectance spectra. Copper phosphate bulks were prepared with a hydrothermal hot pressing method using the powders. The influence of powder conditions on phase formation and bulk properties of the phosphate was studied.

## 2. Experimental

Basic copper carbonate (CuCO_3_·Cu(OH)_2_·H_2_O) was mixed with 85 wt% phosphoric acid (H_3_PO_4_) at a mole ratio of P/Cu = 4:5. Copper orthophosphate, Cu_3_(PO_4_)_2_, was obtained by heating the mixture at 700 °C for 1 hour via the following reaction:

3CuCO_3_·Cu(OH)_2_·H_2_O + 4H_3_PO_4_ → 2Cu_3_(PO_4_)_2_ + 3CO_2_ + 12H_2_O (1)

Due to the volatilization of phosphorus oxide, the mixing ratio of raw materials was settled higher than the P/Cu ratio of the copper orthophosphate. At the same time, basic copper carbonate was mixed with 85 wt% phosphoric acid in a mole ratio of P/Cu = 2:1. Copper *cyclo*-tetraphosphate, Cu_2_P_4_O_12_, was obtained by heating the mixture at 420 °C for 1 hour via the following reaction:

CuCO_3_·Cu(OH)_2_·H_2_O + 4H_3_PO_4_ → Cu_2_P_4_O_12_ + CO_2_ + 8H_2_O (2)

The copper orthophosphate or *cyclo*-tetraphosphate (6 g) was milled with a planetary mill at a speed of 300 rpm for up to 360 minutes, using a Fritsch P7 type planetary mill with five ZrO_2_ balls (15 mm diameter, average weight 8.88 g) and a ZrO_2_ pot (40 mm inside diameter, 40 mm depth).

**Figure 1:**
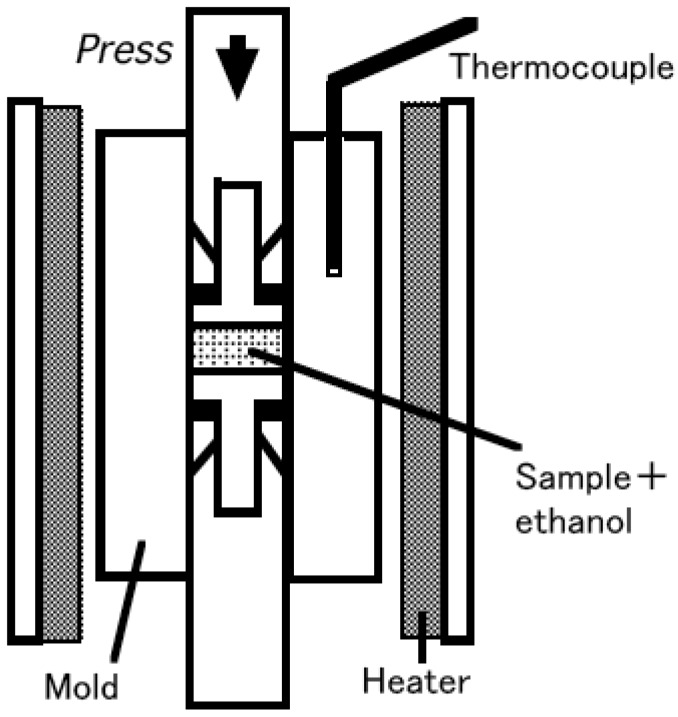
Schematic diagram of hydrothermal hot pressing apparatus.

Figure 1 shows a schematic diagram of hydrothermal hot pressing apparatus. Mixtures of powder and 10 mass % of ethanol were placed in a mold and mechanically pressed with a uniaxial pressure of 40 MPa and subsequently heated at 150 °C at a rate of 10 °C/min for 2 h. In general, water is used in hydrothermal hot pressing process. However, in this work, ethanol was selected, because of concerns that *cyclo*-tetraphosphate would decompose under aqueous conditions. Because the mold had a slight space, ethanol could be volatilized in sintering process. The sintering pressure was determined as general one in hydrothermal hot pressing process.

The un-milled, milled, and sintered materials were analyzed by X-ray diffraction (XRD), and Fourier transform infrared spectroscopy (FT-IR). XRD patterns were recorded using a Rigaku Denki RINT 2000M X-Ray diffractometer using monochromated CuKα radiation. FT-IR spectra were recorded with a Shimadzu FT-IR spectrometer FT-IR8600 using KBr disk method. SEM images of materials were observed using VE8800 from Keyence. Particle size distribution was measured with laser diffraction / scattering particle size distribution HORIBA LA-910. Specific surface areas of phosphates were calculated from the amount of nitrogen gas adsorbed at the temperature of liquid nitrogen by BET method with Belsorp mini from BEL JAPAN. The color of phosphate materials was estimated by ultraviolet-visible (UV-Vis) reflectance spectra with a Shimadzu UV365.

## 3. Results and Discussion

**Figure 2:**
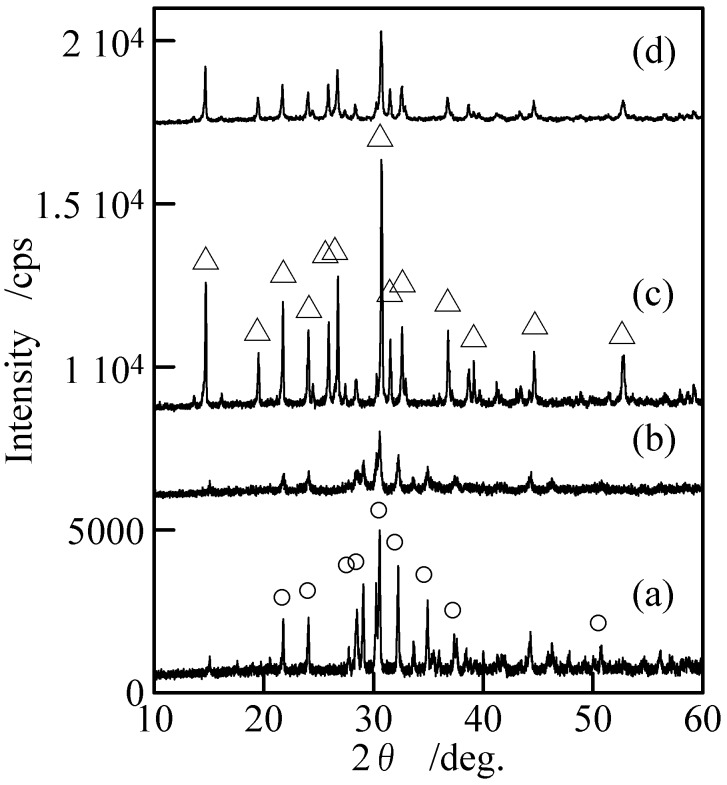
XRD patterns of samples with and without milling, (a) Cu_3_(PO_4_)_2_ without milling, (b) Cu_3_(PO_4_)_2_ milled for 120 min, (c) Cu_2_P_4_O_12_ without milling, and (d) Cu_2_P_4_O_12_ milled for 120 min, ○; Cu_3_(PO_4_)_2_ and △; Cu_2_P_4_O_12_.

**Figure 3:**
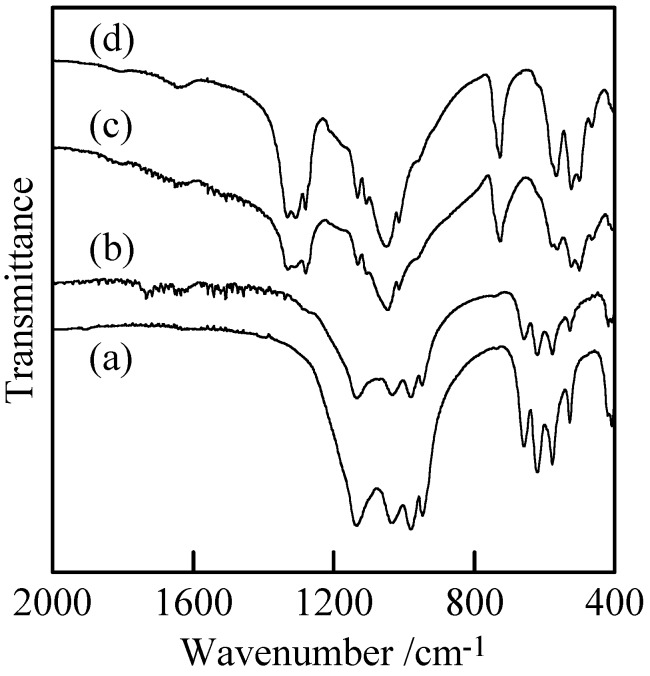
IR spectra of samples with and without milling, (a) Cu_3_(PO_4_)_2_ without milling, (b) Cu_3_(PO_4_)_2_ milled for 120 min, (c) Cu_2_P_4_O_12_ without milling, and (d) Cu_2_P_4_O_12_ milled for 120 min.

Figure 2 shows XRD patterns of the samples with and without milling. Samples without milling [Figures 2(a) and (c)] indicated only the peaks of Cu_3_(PO_4_)_2_ and Cu_2_P_4_O_12_ without any peaks of other compounds. The peaks of Cu_3_(PO_4_)_2_ and Cu_2_P_4_O_12_ became weak after the milling [Figures 2(c) and (d)]. Figure 3 shows IR spectra of the samples with and without milling. The adsorption spectra of the samples prepared in P/Cu = 4:5 and 2:1 can be readily ascribed to those of copper orthophosphate and *cyclo*-tetraphosphate, respectively [Figures 3(a) and (c)] [2,14,17]. These spectra showed no new absorption peaks caused by the milling. The crystallinity of the copper phosphates was weakened by the milling (Figure 2), while the chemical structure of crystalline phase was not changed. Samples after milling contained more amorphous phase than those before milling, because the crystalline phase of copper phosphates were destroyed in the milling process.

**Figure 4:**
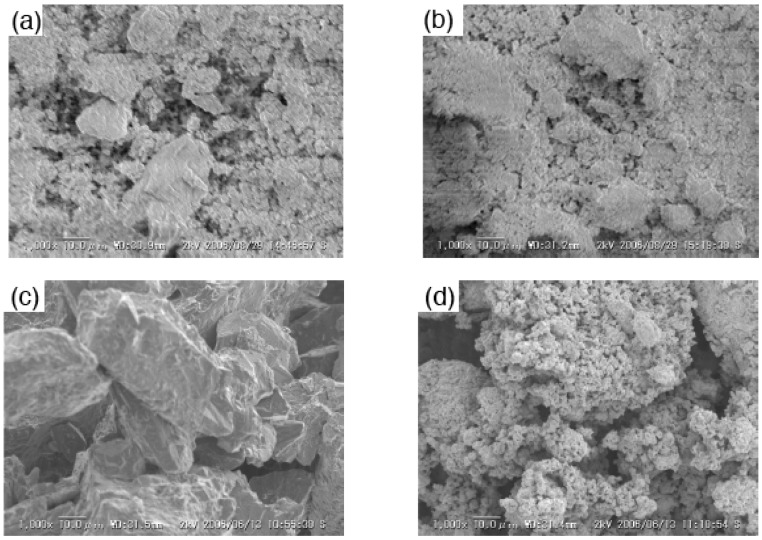
SEM images of samples with and without milling, (a) Cu_3_(PO_4_)_2_ without milling, (b) Cu_3_(PO_4_)_2_ milled for 120 min, (c) Cu_2_P_4_O_12_ without milling, and (d) Cu_2_P_4_O_12_ milled for 120 min.

**Figure 5:**
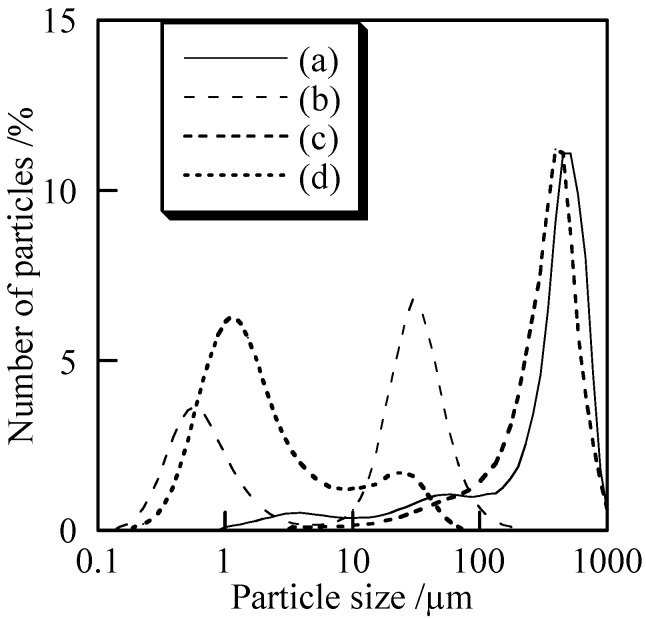
Particle size distribution of samples with and without milling, (a) Cu_3_(PO_4_)_2_ without milling, (b) Cu_3_(PO_4_)_2_ milled for 120 min, (c) Cu_2_P_4_O_12_ without milling, and (d) Cu_2_P_4_O_12_ milled for 120 min.

Figure 4 shows SEM images of the samples with and without milling. All samples indicated no specified shape. The large particles were observed in Cu_2_P_4_O_12_ sample without milling [Figure 4 (c)]. The milled samples consisted of smaller particles than the un-milled ores. Figure 5 shows particle size distribution of the samples with and without milling. Samples without milling had larger particles than 100 µm [Figures 5 (a) and (c)]. In copper orthophosphate, the particle size became much smaller than 100 µm after the milling [Figure 5(b)]. Most particles had sizes smaller than 10 µm in the case of copper *cyclo*-tetraphosphate. Copper *cyclo*-tetraphosphate was more easily refined than orthophosphate, which is probably because *cyclo*-tetraphosphate has high content soft phosphate. 

**Figure 6:**
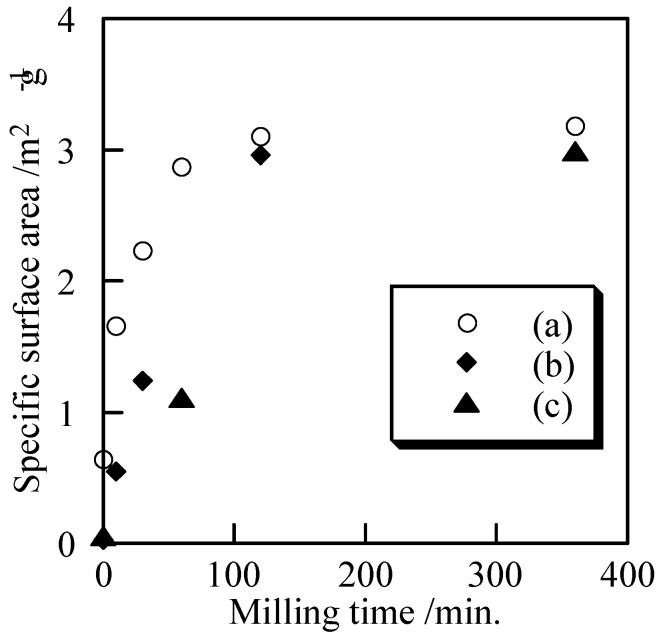
Specific surface area of samples milled for several minutes, (a) Cu_3_(PO_4_)_2_, (b) 1st Cu_2_P_4_O_12_, and (c) 2nd Cu_2_P_4_O_12_.

Figure 6 shows the specific surface area of the samples with and without milling. All samples had small specific surface area, because they were synthesized a relatively high temperatures. Small specific surface area was related with large and non-porous particles. It is expected that high dense bulks are easily formed from small and non-porous powders. Because mechanochemical treatment makes the particle size small, not porous, it is a suitable method to prepare small powders. After milling for 120 min, the specific surface area increased slightly. The specific surface area was not significantly increased after milling for 360 min. The difference of phosphate structure had almost no influence on the samples’ specific surface area.

**Figure 7:**
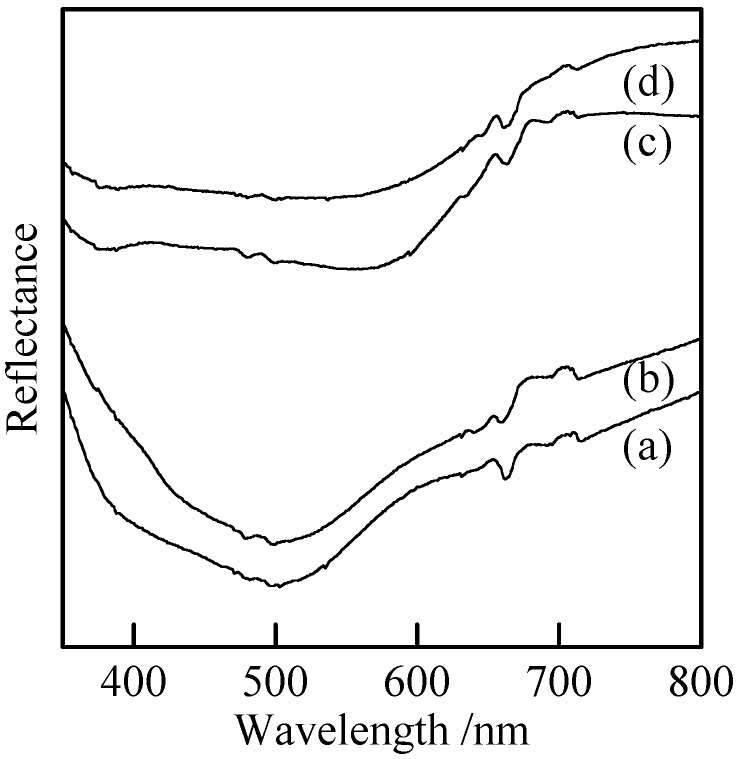
UV-Vis reflectance spectra of samples with and without milling, (a) Cu_3_(PO_4_)_2_ without milling, (b) Cu_3_(PO_4_)_2_ milled for 120 min, (c) Cu_2_P_4_O_12_ without milling, and (d) Cu_2_P_4_O_12_ milled for 120 min.

Figure 7 shows UV-Vis reflectance spectra of the samples with and without milling. The spectrum of Cu_3_(PO_4_)_2_ had a broad peak at 500 nm [Figure 7(a)]. This peak was almost unchanged by the mechanical treatment [Figure 7(b)]. It is also found that particle size the samples did not influence their color very much. Copper *cyclo*-tetraphosphate had broad peaks at 380 and 560 nm in the reflectance spectra [Figure 7(c))]. The hue of this material was not change by the milling [Figure 7(d)]. Further, the hydrothermal hot pressing process had no influence on the color of phosphate bulks.

**Table 1:** Results of hydrothermal hot pressing process on Cu_3_(PO_4_)_2_ and Cu_2_P_4_O_12_ (sample weight: 2 g; solvent: ethanol; solvent volume: 0.2 mL; temperature: 150 °C; heating time: 2 h; pressure: 40 MPa).

Compound	Milling time	Density	Filling factor
	/min	/g cm^-3^	/%
Cu_3_(PO_4_)_2_	0	2.140	53.24
	120	2.520	63.29
Cu_2_P_4_O_12_	0	2.566	74.33
	10	2.819	81.66
	30	2.833	82.07
	120	2.825	81.84

**Figure 8:**
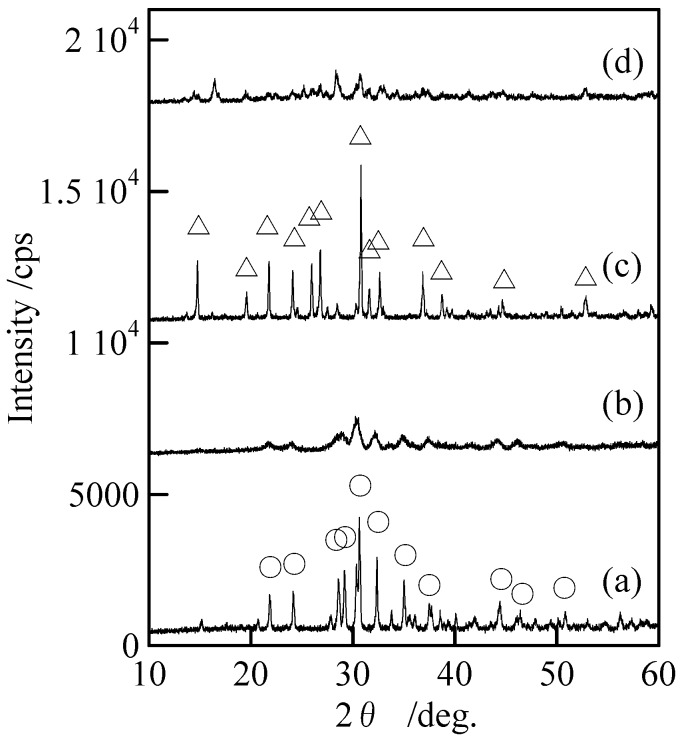
XRD patterns of phosphate bulks, (a) Cu_3_(PO_4_)_2_, without milling, (b) Cu_3_(PO_4_)_2_ milled for 120 min, (c) Cu_2_P_4_O_12_ without milling, and (d) Cu_2_P_4_O_12_ milled for 120 min, ○; Cu_3_(PO_4_)_2_ and △; Cu_2_P_4_O_12_.

Table 1 lists density and filling factor of the phosphate bulks. This filling factor is calculated from the theoretical density of the crystalline structure. Samples derived from the milled powders have higher densities, due to their small particle sizes. The small particle of phosphate material was easy to produce high density of phosphate bulks. Milling time was found to have almost no influence on the density of the phosphate bulks. The density and filling factor of copper orthophosphate were lower than those of *cyclo*-tetraphosphate, which could be due to the soft part of phosphate. Figure 8 shows XRD patterns of phosphate bulks derived from the powders with and without milling by sintering. The samples indicated only the peaks of Cu_3_(PO_4_)_2_ and Cu_2_P_4_O_12_. The vertical scale in Figure 8 is the same as that in Figure 2. The peak intensity in XRD patterns became slightly weak after the hydrothermal hot pressing process (Figure 2 and Figure 8). It was mainly because sintering temperature was too low to increase the samples’ crystallinity and the solvent used might have a negative effect on their crystallinity. Nevertheless, highly dense copper orthophosphate and *cyclo*-tetraphosphate bulks can be obtained by using hydrothermal hot pressing process combined with mechanochemical treatment.

## 4. Conclusions

It is found that mechanochemical treatment can be used to refine copper orthophosphate and *cyclo*-tetraphosphates powders by reducing their crystallinity without changing their chemical structures. With the same milling conditions, copper *cyclo*-tetraphosphate was more easily refined than orthophosphate. Combined with the mechanical treatment, highly dense phosphate bulks have been obtained using a hydrothermal hot pressing process. Copper *cyclo*-tetraphosphate possessed higher density than copper orthophosphate. 
